# Braiding Fruits and Flowers as a Wish of Prosperity and Victory over Death in the Carved Festoons of Ancient Rome

**DOI:** 10.3390/plants13192795

**Published:** 2024-10-05

**Authors:** Alessandro Lazzara, Alma Kumbaric, Agnese Pergola, Giulia Caneva

**Affiliations:** 1 Department of Science, University of Roma Tre, Viale Marconi 446, 00146 Rome, Italy; alessandro.lazzara@uniroma3.it (A.L.); alma.kumbaric@uniroma3.it (A.K.); 2 Museo Nazionale Romano, Largo di villa Peretti 2, 00185 Rome, Italy; agnese.pergola@cultura.gov.it; 3 NBFC, National Biodiversity Future Center, Piazza Marina 61, 90133 Palermo, Italy

**Keywords:** plant symbolism, plant history, Roman culture, Roman archaeology, phytoiconology, Roman festoons, Roman wreaths

## Abstract

Plant motifs had a significant role in ancient cultures, with decorative, artistic, and communicative values. However, little knowledge exists of the botanical composition of festoons, widely used in Greek-Roman art. We analysed 81 festoons, exclusively from sculpture artworks, collected from 13 museums and archaeological sites in Rome (1st century BC–3rd century AD). Using iconographic sources and previous data, we identified the represented species and analysed their abundance and composition using statistical methods (Cluster Analysis, Principal Components Analysis) and diversity indexes (Shannon and Evenness). We documented 3081 botanical elements, identifying 30 taxa, in which fruits with leaves (45%) or alone (10%) represented the most common ones. *Laurus nobilis* and *Quercus* cfr. *robur* were the most frequently depicted species, followed by “pomes” (*Pyrus*, *Malus*, *Cydonia*), *Vitis vinifera*, *Punica granatum*, and *Ficus carica*. Festoons with one or two species can be easily distinguished from those with multiple species, often arranged with figs or vine branches at the ends, with symbolic aims related to fertility, rebirth, and abundance values. A balanced botanical composition also exists, with flowers typically in the middle and a species distribution that is not casual. The results enriched our comprehension of ancient Roman society, considering funerary and celebrative events.

## 1. Introduction

In ancient times, all around the world, natural elements played a central role in different aspects of human daily life, such as medicine and food, as well as in the cultural and religious sphere [1,2,3,4,5]. Ancient societies were directly in contact with nature, where different biological species, like plants and animals, and various meteorological and geological phenomena assumed importance in both the practical and the symbolic sphere [1,5,6]. Being unable to explain the scientific reasons behind the natural phenomena, ancient people interpreted the natural shapes and phenomena considering the presence of divine entities and by the creation of myths [2,7,8,9]. 

The representation of plant motifs, which is commonly found in different kinds of archaeological artworks, assumed an important role in ancient cultures, such as among the Egyptians [10,11], the Assyrians, the dynasty of Achaemenids in ancient Persia [12,13], in the Mesopotamian area (i.e., during the Uruk period) [14,15,16], the Etruscans [17,18], and the Greek–Romans [6,7,19,20,21]. The representations of nature assumed various forms and symbolic power according to the context of representation, as well as the position or repetition of natural elements [22,23]. Then, such representations not only had a decorative or artistic value but rather constituted a non-verbal communicative tool or an expression of the deities will [23,24,25,26]. 

Several ancient authors, in their surviving poems and literary works, highlighted the sacred value of nature in the Greek–Roman culture, but considering the loss of much ancient documentation (i.e., *De Plantis* attributed to Aristotle, some parts of the writings of Theophrastus and other minor authors), artistic elements can be helpful in tracing the cultural and religious relevance of nature [27,28]. The representation of nature is attested in many types of artworks [29], such as in the frescos of gardens, where plants have been depicted with high accuracy [30,31,32], in the so-called “idyllic landscapes” [33], and also in the carved sculptures of monuments [21,34], mosaics [35], and architectural stones and terracotta [36]. The external friezes of the Ara Pacis of Augustus (the monument to peace) in Rome, where more than 90 different plants are represented, feature single elements and details that assume an extraordinary richness and fidelity, which are outstanding demonstrations of the knowledge of and value of nature, and of its use for communicating messages [37,38,39]. Furthermore, the presence of plants is also particularly evident in wreaths, garlands, and festoons, which were frequently represented in Roman art as decorations in altars (funeral or sacral), generally flanked by cupids, sacrificial animal skulls or mythological scenes, or reproduced as frescos in domestic spaces [28,40,41]. Wreaths, which are sometimes found in archaeobotanical remains [39,40,42,43], were an arrangement of flowers, leaves, or stems fastened in a ring and used for decoration or laying on a grave. They were called garlands when used as a mark of honour or victory. Festoons, instead, were made of fruits and flowers, tied with ribbons, and suspended at both ends in a loop [44]. The word “festoon” is derived from the Latin word “*fĕsta*”, i.e., “festive, solemn”, for their use during rituals and celebrations.

The tradition of using intertwined plants in festoons and garlands, with a clear function and a symbolic aim [41], can be traced back to ancient cultures, including the Egyptian, where they were used for the adornment of mummies; or Persian and Etruscan civilisations, where they were also used in kings’ dresses as symbols of beauty and power [18,43,45]. Later, Mediterranean civilisations acquired the same tradition, in particular during the Roman period, when the manufacturing of overall flower garlands and festoons became a real business in Roman society [30]. Information on garlands comes from Pliny (*Naturalis Historia (NH)*, 21.4) [46], where he describes their manufacture, how they changed through time and, in the end, which flowers were used for their realisations according to the season [30]. However, little is still known about the plant composition of festoons in the ancient Mediterranean context, particularly in the Roman world. A detailed study of the floristic diversity in the Roman festoons has not yet been made, and a deeper investigation is needed to give an accurate view of their composition, as well as of their symbolic and cultural value. Considering the role that they played in Roman society, especially in funeral and religious contexts, and to better comprehend the role of the different plants, this study, which is limited to carved artworks, aims to define: (i) the species and related parts that were associated with the carved festoons in Roman art, such as their recurrence and their specific significance and symbolism; (ii) the combination and variability of botanical elements and their organisation, establishing possible changes associated with the chronology, context, and typology of the artworks.

Then, by investigating the festoons’ plant diversity, the recurrence of botanical elements and their symbolic value from various artworks of different ages collected from Museums and Archaeological Parks of Rome, and applying statistical tools, we will enhance the botanical richness and the selection and disposition of the various species. This will enrich our comprehension of ancient Roman society and support the understanding of archaeobotanical remains from archaeological excavations.

## 2. Results

**Figure 1:**
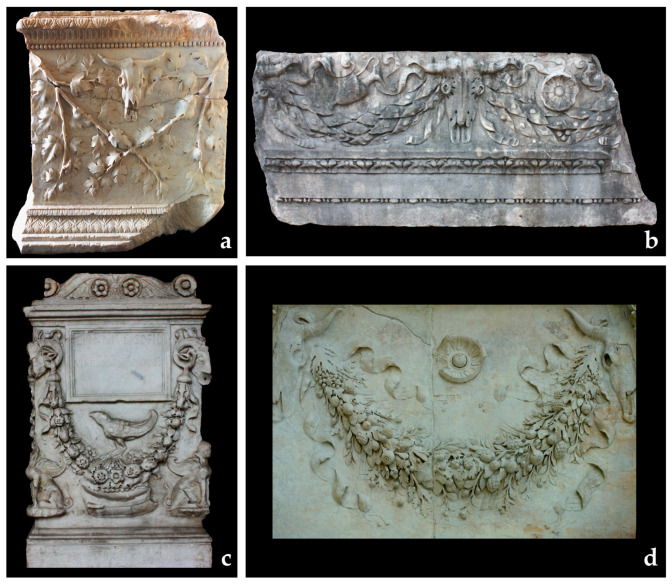
Examples of mono-specific (**a**,**b**) and multi-specific (**c**,**d**) festoons in different typologies of artworks. (**a**) Base (inventory number 417) from the *Museo Nazionale Romano* (PM-8, INV. 417); (**b**) frieze fragment from the Diocletian baths (TD-82, INV. 78137-78139); (**c**) funerary altar of *Lucius Pinnius Celsus* from *Palazzo Altemps* (PA-3, INV. 8599 bis); (**d**) festoon from the internal monument of Ara Pacis. (Photos by the Authors,—Courtesy by the Ministry of Culture, *Museo Nazionale Romano*).

**Figure 2:**
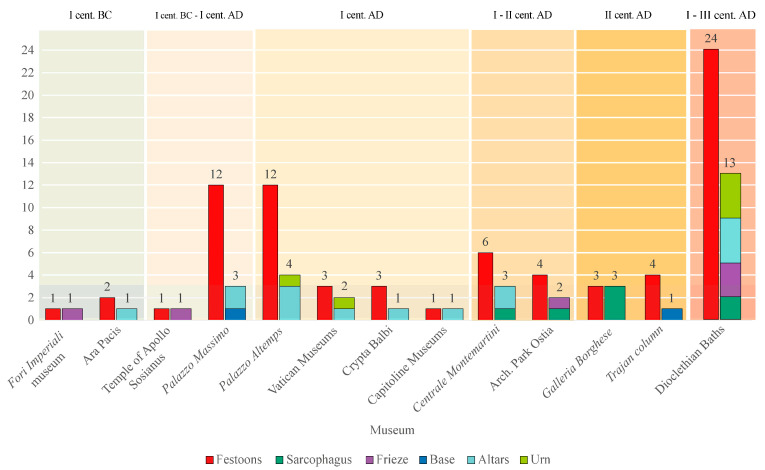
The analysed festoons of the considered Roman museums and archaeological parks listed in chronological order, showing the number of festoons (first column) from each, the number of artworks and the correlated typology (second column) where the festoons are represented.

A total of 81 festoons was collected from 36 artworks belonging to 5 different typologies (altars, bases, friezes, sarcophagi, urns) that can be included in 4 different contexts (Figure 1 and Figure 2). The most recurrent context was the funerary (69%, seven sarcophagi, six urns, 12 altars); then the sacred (19%, three altars, one base, three friezes); and the celebrative (3%, one base), over the unknown (8%, three friezes). Thirty-three festoons were in a very good preservation status, 24 of them in a medium status, and the remaining 24 were in very low conservation status that sometimes compromised the botanical identification (see Supplementary Data, S1a). In a few cases, restoration activity and the substitution of elements were carried out, generating some doubts regarding the interpretation of plants.

At first, it was possible to distinguish two main categories of festoons (see Figure 1): the mono-specific-festoons (29 festoons with only one or a maximum of two species, mainly leaves and related fruits) and the multi-specific-festoons (52 festoons enriched by the fruits, leaves, and flowers of many species). 

Mono-specific festoons can be present alone or in combination with multi-specific ones. In such cases, they were often placed at the sides of the altars (where their presence is mainly recorded), in association with the *patera* and the *uraeus*, as common religious instruments in ceremonies. Sometimes, the species can be represented in well-visible branches with leaves (Figure 1a) or only by leaves and fruits that hide the branches (Figure 1b).

### 2.1. Identified Plants, Recurrence and Their Symbolism

In the 81 analysed festoons, we detected 3081 sculpted botanical elements, and we successfully identified 2357 elements belonging to 30 taxa of Gymnosperms and Angiosperms, including 21 families, 26 genera, and 23 species. The remaining 724 elements, because of the low preservation status of the artworks, i.e., significantly damaged or missing elements, were unidentifiable. The exact number of species is uncertain, and it could eventually increase due to some similarities between species or the absence of morphological diagnostic details. In these cases, it was often possible to define the elements only at the genus or family level. Some of the main cases in which identification was more complex were for *Pinus* and *Quercus* representations, where we sometimes attributed the element to a specific species and in other cases we indicated only the genus. Similarly, especially in case of a bad state of preservation, we considered a “pomoid shape”, categorising them as “NN POMOID”, meaning an ambiguity in the *Malus* and *Pyrus* genera and *Cydonia oblonga* species, but without a certain attribution. Nonetheless, we do not exclude the possible presence of varieties of apple and pear (such as *Malus sylvatica* or *Pyrus piraster*), but for the absence of diagnostical details, we decided to maintain them in a single species (*Malus domestica* and *Pyrus communis*).

Table 1 shows the botanical taxa identified in the analysed festoons, enhancing the represented plant element, their chorology, diagnostic traits and eventual possible confusions, and, finally, their symbolic value in Greek–Roman cultures.

**Figure 3:**
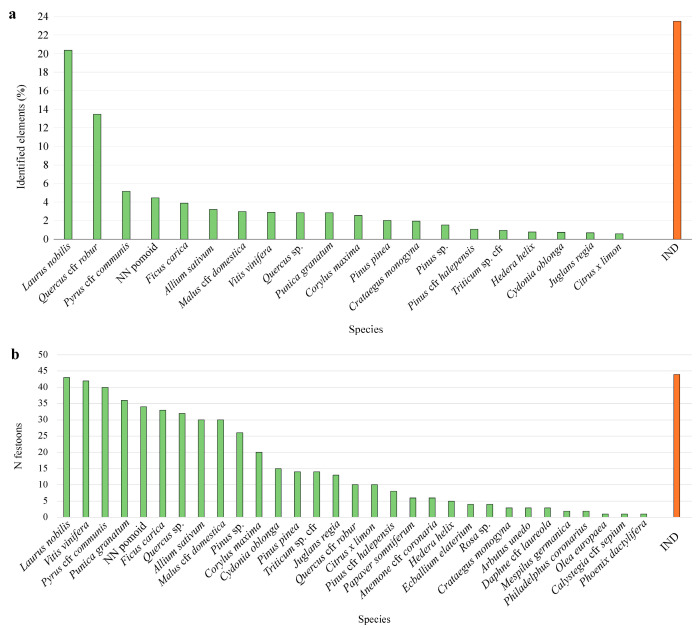
(**a**) Relative frequency of the species among the festoons, considering the values higher than 1%; (**b**) occurrence frequency of the species, i.e., number of festoons presenting them.

In most cases, the parts of the represented plants were fruits with the corresponding leaves (49%), or fruits alone (10%), leaves (11%), cones (5%), bulbs or roots (3%), inflorescences and flowers (both 1%), and one element representing an entire plant. In 23% of cases, we found unidentifiable elements (see Figure 3) (e.g., the flowers in very low preservation status were distinguishable only according to the general shape and the number of represented petals (4-petaled (2 typologies), 5-petaled (2), 6-petaled (4), and 3-petaled (1) flowers). In some cases, such as for *Citrus x limon* and *Philadelphus coronarius*, we have some doubts about the original presence in the ancient Roman festoons due to the occurrence in widely restored artworks (e.g., F-PA 7).

The identified species showed a different frequency and recurrence among the 81 festoons (Figure 3), with *Laurus nobilis* and *Quercus robur* showing both leaves and fruits, also widely occurring in the mono-species festoons that constituted 628 and 415 of the total elements, respectively. Identified plants with a relative frequency under 1%, excluded from Figure 3a, were mostly flowers (*Ecballium elaterium*, *Rosa canina*, *Daphne* cfr. *laureola*, *Anemone coronaria* cfr., and sometimes fruits (*Arbutus unedo*, *Mespilus germanica*)).

When considering the presence in the different festoons (Figure 3b), laurel leaves with fruits also resulted as the most recurrent plant element, being present in 43 festoons, followed by fruits and leaves of *Vitis vinifera*, which mostly recurred at the initial parts of the festoons. Other taxa, such as *Punica granatum*, *Pyrus communis*, *Allium sativum*, *Malus domestica*, *Quercus*, and *Pinus*, were also highly recurrent, being present in about half of the analysed festoons. 

**Figure 4:**
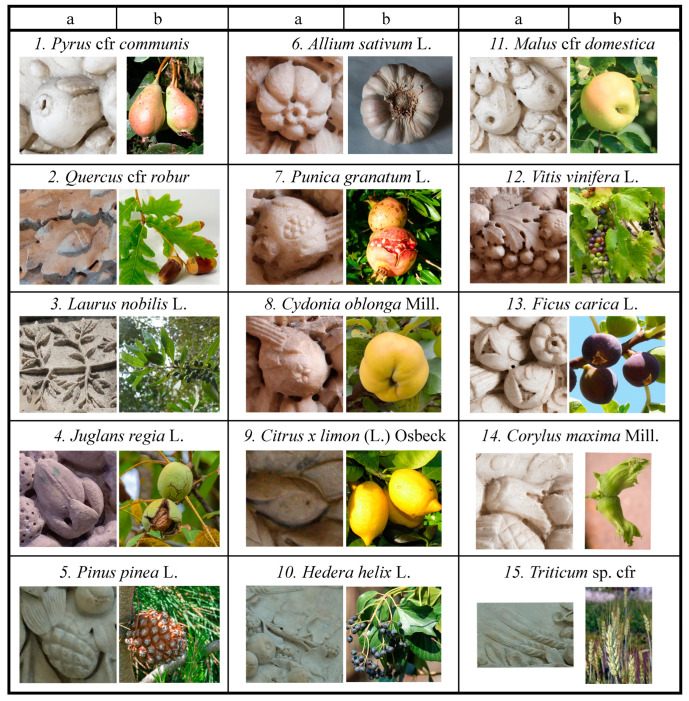
The most recurrent botanical elements in the festoons (**columns a**) compared to correspondent fresh plants in nature (**columns b**). Photos of the carved elements by the authors, except for n. 1, 2, 11, 13, 14a (www.collezionegalleriaborghese.it accessed on 6 December 2023); (**a**): 1, 11, 13, 14a from Festoon F-GB1; 2a from F-CB1; 6, 7, 8, 12a from F-TD 108; 5, 10, 15a from F-AP1; 9a from F-PA 7; 4a from F-CMM3; 3a from F-TAS. (**b**) Pictures of fresh plants: 1, 7, 9, 10, 11, 12, 14, 15b (from https://dryades.units.it/cercapiante/index.php accessed on 30 March 2024), 5, 8, 3, 12, (GBIF, Global Biodiversity Information Facility, https://www.gbif.org accessed on 30 March 2024), 2b, 4b, 13b (Acta plantarum, https://www.actaplantarum.org/ accessed on 30 March 2024), 6b (photo by the authors).

Figure 4 gives some iconographic examples of the most recurrent species, enhancing their similarity with elements in nature.

Most species are strictly located in the Mediterranean basin (*Olea europaea*, *Arbutus unedo*, *Laurus nobilis*) or in the Euri-Mediterranean area (e.g., *Pinus pinea*). Euro–Asian (e.g., *Crataegus monogyna*) and Asian species (*Allium sativum*, *Cydonia oblonga*, *Punica granatum*) were also well represented. More rarely, some species also came from the subtropical (e.g., *Phoenix dactylifera*), the subMedit-subAtlantic (e.g., *Hedera helix*), or the Mediterranean–Turanian area (e.g., *Ficus carica*).

As reported in Table 1, the represented taxa had a different symbolism, and the choice of representing fruits expressed the idea of nature’s abundance and prosperity. In general, a similar symbolic aim seems to occur in all the analysed festoons without relevant changes associated with the artworks’ chronology, context and typology.

Whereas *Laurus nobilis* symbolised victory over death, as a sign of eternity, being the emblem of Apollo, the God of the Sun (Pliny, *NH* 12.2), *Quercus* cfr. *robur* represented strength, longevity, and fertility, making it suitable to be associated with the father of the gods, Zeus (Jovis). A relation with the God Zeus/Jovis is also expressed by the common presence of *Juglans regia* and *Corylus maxima*, while *Ficus carica* is symbolically linked to the God Mars and the myth of the foundation of Rome.

Most of the other taxa were strongly linked to concepts of fertility and fecundity and related to the idea of feminine and maternal elements, such as *Triticum* sp., *Pinus* sp., *Punica granatum*, *Cydonia oblonga*, *Malus domestica*, and *Pyrus communis*. A significant role was also observed for the Dionysian symbols, which were related to the concept of vegetative strength (i.e., *Vitis vinifera*, *Hedera helix*, *Calystegia sepium*, *Ecballium elaterium*). Moreover, the victory over death can be enhanced by *Arbutus unedo* and *Anemone*, which embodies immortality and rebirth. Other species, such as *Allium sativum*, had a relevant significance mostly according to their superstitious value, from which their relevance as salvific plants could be derived. 

### 2.2. Combination and Variability in the Structure and Organisation of Festoons 

**Figure 5:**
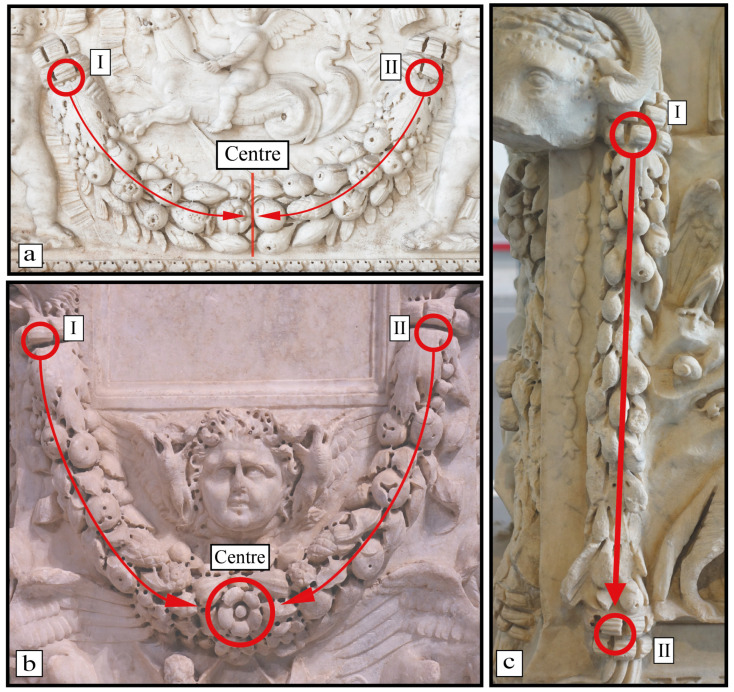
General scheme of the organisation of Roman carved festoons. (**a**) Festoon with semicircular shape with the central area which divides the festoons into two halves, without a central botanical element (n = 43) as in the sarcophagus with festoon with heroes and marine motifs from the *Galleria Borghese* (F-GB 3); (**b**) festoons composed by a central area and a central botanical element (n = 30) as in the Circular Altar *from Centrale Montemartini* (F-CMM 4); (**c**) the anomalous festoons in the Altar dedicated to Mars, Venus, and Silvanus from *Palazzo Massimo* (F-PM 9) with a vertical distribution of the elements, without a central area (n = 8). (**a**) Courtesy by *Galleria Borghese* Museum, www.collezionegalleriaborghese.it accessed on 6 December 2023); (**b**,**c**) photo by the authors—Courtesy of the Ministry of Culture, *Museo Nazionale Romano*).

The study of the festoon’s structure led to sequencing all 81 festoons. During this process, we noticed some general structural differences between the festoons and how the plant botanical elements were arranged. Firstly, we figured out that the carved elements are mostly oriented from the starting and the ending points toward the central part of the festoon. Moreover, the festoon shape is usually semicircular, as an arch made from pending elements starting from cupids or heads of goats, bucrania, or panthers (Figure 5a). Sometimes, the centre is not defined by certain species, whereas sometimes, a central botanical element arises (Figure 5b). The only exception is given in the artefact PM-9, where the eight festoons have a vertical distribution, going from the upper to the bottom extremities, without a central area (Figure 5c). 

**Figure 6:**
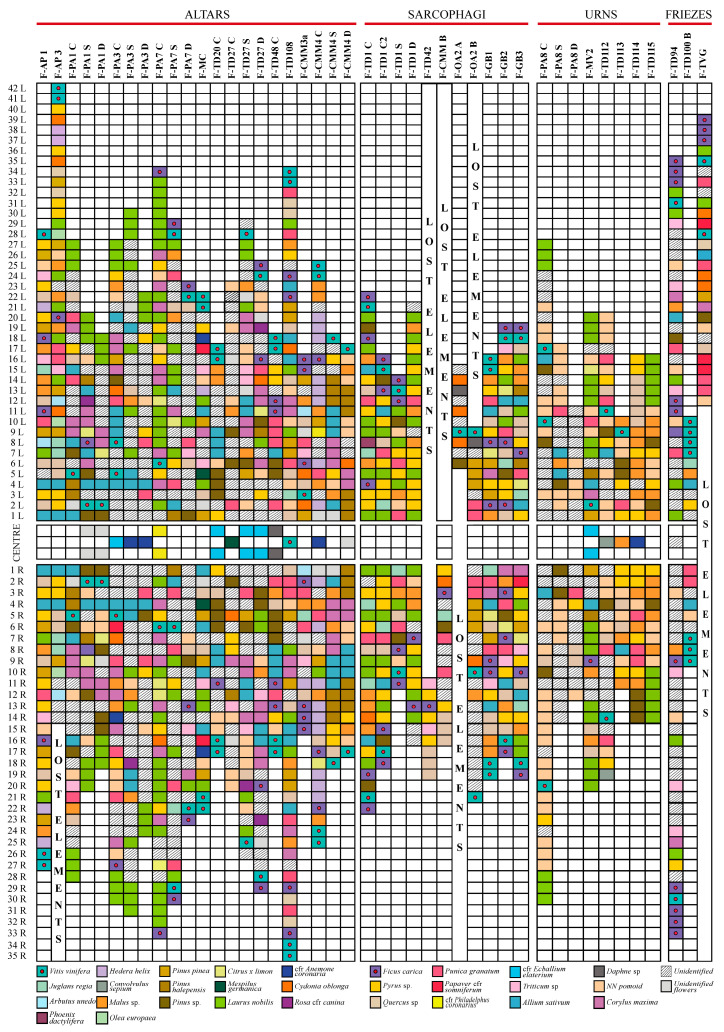
Examples of sequences of plants (see the legend for colours) in the four groups of typologies (Altars (I), Sarcophagi (II), Urns (III), Friezes (IV)) of festoons with central elements (n = 44) (numeration starts from the centre until the two ends). The festoons from artwork PM-9 have been excluded for their different structure organisation and the low state of preservation.

The sequences of the multi-specific festoons were used to analyse the variability of the organisation of the botanical elements (Figure 6).

This analysis enhanced some relevant points about the disposition of species in the multi-specific festoons (n = 52). For example, the fruits and leaves of *Ficus carica* and *Vitis vinifera*, which highly recur in the festoons (see Figure 3b), are mostly located at the beginning and the ending points (37% and 23%, respectively). The remaining 40% of them are occupied by leaves of laurel (21%), oak (4%), pomoid-shaped fruits (10%), or unidentified elements (6%) (see Figure 6). Vine grape and fig can be found represented in different combinations (leaves and fruit of vine grapes; leaves of fig alone; leaves of fig alone; followed by grape).

Moreover, we can also observe that usually, the number of species present in the festoons can vary, with a maximum of 17 species detected in two festoons (F-TD 27D and F-AP 3) and a minimum number of 4 species, which is more frequent across the considered festoons, and this may be correlated to the high number of unidentifiable or missing elements. In general, it’s possible to assess an average of 10 ± 4 botanical species in the festoons.

The diversity indexes (Shannon and Evenness), calculated for the multi-specific festoons in relation to their typologies and contexts, enhanced some statistically significant differences between the groups (Table 2). In particular, some differences emerge according to the contexts and the chronology, both in terms of Shannon and Evenness indexes (*p*-value < 0.05). Contrarily, regarding the typologies, such tests did not present statistically significant plant diversity and the equal distribution of species. Indeed, Tukey’s post-hoc test showed that Funerary and Sacral were the only ones showing significant differences for both Shannon and Evenness (*p*-value < 0.05), while for typology, the differences resulted not significant, even if between the Unknown and Sacral contexts, the values were slightly higher than the threshold value (Table 3). For chronology, the Tukey test showed significant differences between the festoons dated to I–II century AD and those belonging to other chronological periods (Supplementary Data, S1b). 

**Figure 7:**
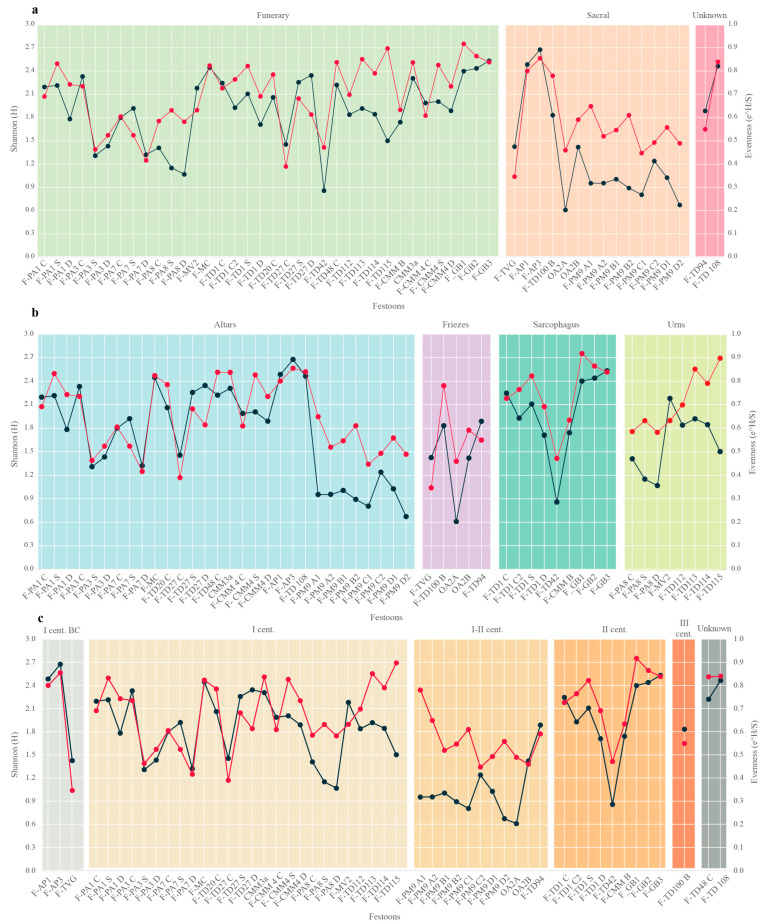
Trends of Shannon (Black line) and Evenness (Red Line) indexes of the multi-specific festoons, related to the plant diversity values across the festoons, in relation to: (**a**) the context of provenance (Funerary, Sacral, Unknown); (**b**) the typology of artefacts (Altars, Sarcophagi, Friezes, Urns); (**c**) the chronology.

In Figure 7, the values of Shannon and Evenness and their trends are represented in relation to contexts (7a), typologies (7b), and chronology (7c). 

Regarding the contexts (Figure 7a), the results showed a significant variability of species in the festoons, with several fluctuations characterised by both low and high values for both the Shannon (range between 0.61 to 2.68) and Evenness (0.3–0.9) indexes. 

The funeral festoons (n = 36) present a Shannon index that ranges between 1.7 and 2.53, except for sample F-TD42 (H = 0.47). This festoon is characterised by a high number of unidentified species, which probably contributes to its low plant diversity. The festoons from sacral contexts (n = 11) vary particularly in number of species, including both the highest (F-AP, H = 2.68) and the lowest (F-OA2A = 0.61) values of Shannon of all samples, with a variability (1SD = ±0.6) more enounced than in the other contexts (1SD = ±0.4). The presence of samples showing the lowest value of plant diversity across the whole dataset could be associated with the inclusion, in the sacral samples, of some of the festoons with highly unidentified species (i.e., F-PM-9). The remaining values from unknown contexts are comparable to those from funeral and sacral contexts (Figure 7a). 

When analysing the different typologies of festoons, similar trends are still visible, with variable values of Shannon and Evenness indexes (Figure 7b). In the altars (n = 30), the variability of both indexes is more enounced, with an average range between 1.15 and 2.35 (calculated as mean ± 1SD), while for the other three typologies (urns, sarcophagi, and friezes), the variability of plants into the festoons is lower, with sarcophagi and friezes with 1SD = ±0.5, and urns with 1SD = ±0.4. This difference can be related to the larger number of festoons coming from altars than other typologies. 

Regarding possible chronological distinctions, as already observed for contexts and typology, trends of Shannon and Evenness of festoons showed a correlation, growing and decreasing proportionally in the considered chronological period (Figure 7c). There are some exceptions, including the I century AD, from which most of the samples are dated (n = 26) and the period between the I and II century AD, where, in some samples, a high evenness corresponds to the low diversity of plant composition. As previously mentioned, this difference can be related to the preservation status and the high number of unidentified elements in the considered festoons. In general, the Shannon and Evenness indexes show a correlation in all the considered variables, with the number of species present in the festoons mostly varying proportionally to their equal distribution.

**Figure 8:**
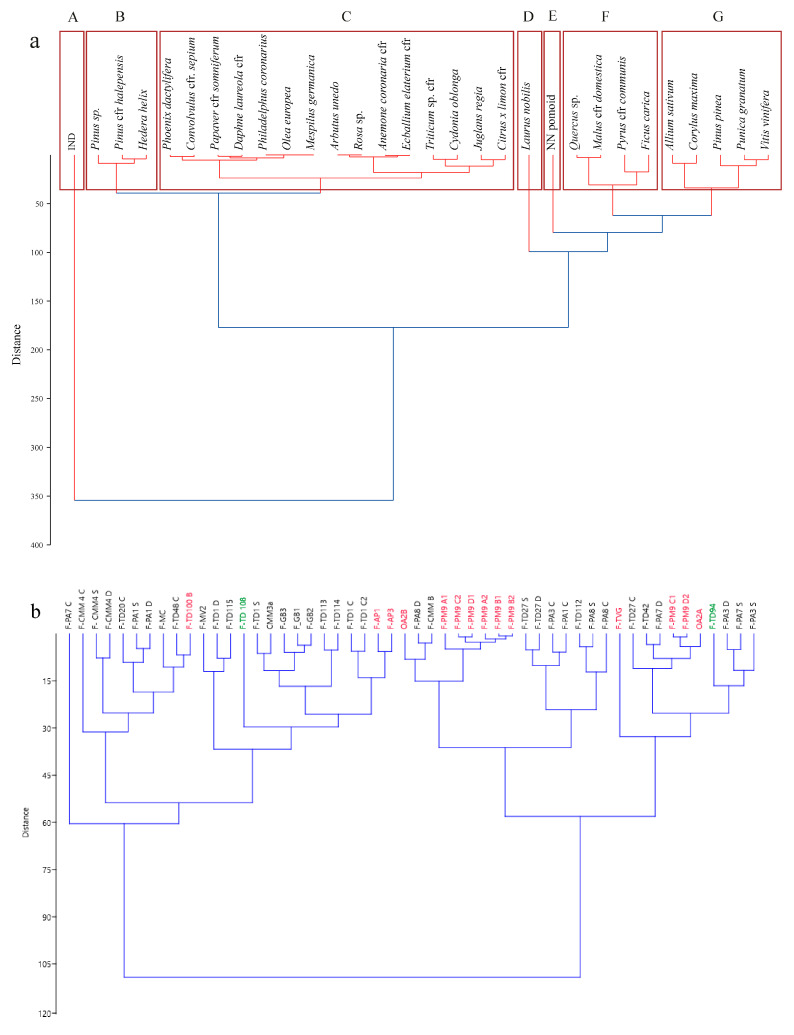
(**a**) Cluster analysis showing the similarities of species in the different festoons; (**b**) similarities of festoons according to their botanical composition considering their context of provenance (red = sacral, black = funeral; green = unknown).

The cluster analysis of species indicated some similarities, showing seven main clusters (marked in red in Figure 8a). Group B, which includes all the flowers found among the festoons, is the widest, while groups A, D, and E are composed of single species or unidentified elements (Group A overall by unidentified elements, while D and E are *Laurus nobilis* and NN Pomoid, respectively). 

Moreover, the cluster analysis of the festoons according to their botanical composition (Figure 8b) enhances the presence of two main clusters composed of festoons from sacral and funerary contexts. It indicated the similarity of species, meaning that the choice of species is independent of the contexts, and the festoons of unknown contexts also appear to be partially associated with the funerary context (F-TD94) and with both contexts (F-TD108).

**Figure 9:**
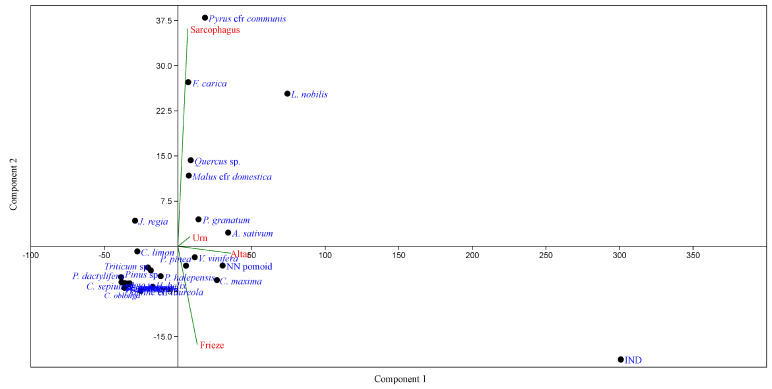
PCA of the species in relation to the typologies of artefacts (Urns, Altars, Sarcophagus, Friezes).

The similarities of the pattern of species in the festoon are also enhanced by the results of PCA (Figure 9), which shows that the majority of the species seem to be common without any relation to a specific type of artwork. The only exception is given by *Pyrus communis* and *Ficus carica*, which appear to be more related to the typology of Sarcophagi. 

## 3. Discussion

The analysis of shapes and compositions of archaeological material gives the opportunity to obtain further information about ancient beliefs, religions, and rites [26,27]. Nevertheless, the symbolism of images is not always easy to interpret, considering that their representation and meaning could change for social or political reasons, the interactions of different cultures or according to the historical context [40,66]. This is also true for plant motifs and their connection to Greek–Roman mythology, which can be complex and is, still nowadays, a debated topic. However, recent studies [6,21,31,32,34,37,39,48,67,68], thanks to the analysis of ancient written sources and pictorial and archaeological artworks, underlined some recurrent connections between certain plants and mythology and, as a consequence, their symbolism.

When looking at the festoons, we know that in Roman society, they were used in several different daily-life occasions, such as festivals, weddings, and banquets, or as symbols of new life when placed in a funeral or religious architecture, becoming one of the recurrent botanical motifs which can be found in ancient artworks [28,41,43]. Furthermore, they were often used as offerings for the dead in funeral rituals [69], fitting our broad recurrence of funerary artworks, particularly altars, followed by sarcophagi and urns. 

The monospecific festoons, mainly composed of oak and laurel, follow a shared tradition as wreaths in both Greek and Roman cultures, recurring in rites and celebrations [41]. Laurel leaves and branches started to be used as crowns for the coronation of Emperors after military accomplishments, particularly in the Augustan age [56], while oak was considered the most sacred tree of all [52,54], used in the making of *Corona Civica*, a crown given to soldiers who accomplished relevant actions in favour of other citizens or against enemies, as Pliny reports (*NH*, 16.3). Moreover, the representation of mono-specific festoons as branches with leaves (of laurel in F-TAS and *Crataegus monogyna* in F-PM-8), as they can be found in a wild landscape, recalling the sacred groves that represented the first typologies of temples where one could connect with the deities (Pliny, *NH*, 12.2) [1,18,46,54,70,71].

A particular change was already observed regarding the combination and variability of the botanical elements and their organisation. In the Hellenistic period and the first imperial period, festoons were composed of leaves of just one species, while later, the richness of botanical species in festoons grew, including fruits and the combination of more species [72]. 

Some general assessments in terms of plant diversity in the structure according to typology, contexts, and chronology can be done. The festoons showed, in fact, a variable plant composition which seems to depend mainly on the context in which they are represented. Despite the lower number of samples, the sacral context shows a higher variability of plant diversity, including the richest and poorest festoons of the dataset. Typology and chronology, conversely, probably affected the choice of species and their abundance in the festoon’s representation in a less significant way. In all cases, such values show a similar trend, growing and decreasing proportionally. Moreover, across all contexts, most of the festoons showing low Shannon values and high evenness values are either characterised by a high presence of unidentified elements (F-TD42, F-OA2A, F-PM9, F-PA8S, and C) or miss some portions due to their preservation status (PM-9 A1, A2, B1, B2, C1, C2, D1, and D2), which may contribute to a reduced Shannon index. The same differences emerge when looking at the typology and chronology that could be related to the abundance of unidentifiable elements in certain festoons (friezes and those dated between the I and the II century AD). In addition, the festoons coming from the funerary context, altars, and the I century AD are more frequent than the other categories.

Considering the botanical elements associated with festoons in Roman art, such as their recurrence and specific significance and symbolism, we believe that the selected species contributed to giving augural messages. The prevalence of fruits found in the festoons can be explained by considering that they were a symbol of deities’ gifts, also in relation to their several beneficial uses [54,73,74]. Moreover, the predominance of fruits can be related to the ripening of nature and the eternal life of the earth [75,76,77], making them also suitable in both funerary and sacral contexts. In fact, the identified species show various symbolic values and usages, giving connection with the marriage ceremonies and the related goddess Aphrodite/Venus (e.g., *Malus domestica*, *Punica granatum*, and *Crataegus monogyna*) [49,52,57,64], or to the maternal and feminine deities, like the Great Mother Cybele/Hera/Juno (e.g., *Pinus* sp., *Cydonia oblonga*, *Pyrus communis*) [22,52,53]. Further connections to the God Zeus/Jupiter (*Corylus maxima* and *Juglans regia*) [39,51] and to Dyonisus/Bacchus, as symbol of vegetative forces (*Ecballium elaterium* and *Hedera helix*) [39,48,54] are also present. Moreover, all of them share a common symbolism of fecundity and fertility, which is represented and recurrent in a large portion of the analysed festoons. The plants linked to these concepts recur with different frequencies, some higher (i.e., *Pyrus*, *Malus*, *Corylus*, *Pinus*, *Laurus*, *Punica*) than others. Still, it is not possible to detect recurrent patterns linked to typology or context. However, we can assess that the central area of the festoons is generally occupied by flowers (Figure 5), which, according to Pliny (*NH*, 21), were extremely important in ancient societies, as they were used in numerous religious and funerary rituals explaining their central role in the festoons. Moreover, these flowers also had relevant symbolic meanings, such as *Anemone coronaria* or *Papaver*, which were related to the concepts of rebirth and prosperity, respectively [47,48,49,61]. The presence of these species and their combination among the festoons highlight augural messages of prosperity and immortality, with a surprising absence of poisoning (e.g., oleander) or death-meaning plants (e.g., spruce) [22].

According to McCann [69], starting from the I century AD, particularly during the Hadrian reign (117–137 AD), the immortality of the dead souls became an increasingly central concept in Roman funerary beliefs. This suggests that the represented species were not casual but probably chosen in relation to their symbolism. 

In addition to the importance of the species previously mentioned, the leaves and fruits of figs and grapevine may have played an important symbolic role, as they characterise the beginning and the end of most of the festoons. The first position of these plants lets us hypothesise that the construction of the festoons could be based on their branches, on which the other botanical elements were added. In Roman culture, as in Greece, grapevine and figs were linked to the cult of Dionysus, present in Rome from at least the II century BC, and known as Bacchus [78]. Dionysus/Bacchus were the deities who, among all, were more linked to vegetation, considered as the divinity of the lymph—the ‘blood of plants’—which rises from the earth every spring and resuscitates the trees [65]. Dionysus symbolised fertility and the secret forces of nature, but also of fertilising pollen and the nectar of flowers, and in the animal world, he reigned over the equivalent fluids, which are blood and sperm. In Greek rites, the wine festival was also a funereal ceremony that renewed the union between the son god and Mother Earth in the natural cyclical spring awakening, having a connection with Persephone in the Eleusinian festivals [65]. Dionysus, like Osiris, the god of death and the afterlife in the Egyptian context, was a divinity torn to pieces and thrown to the ground, who sacrifices himself for everyone, dying and then being reborn, and its representation was also used to wish for resurrection and to symbolise rebirth [79].

Furthermore, in Rome, Dionysus was merged with the pre-existent god of wine and fertility, *Liber*, which led to a complex fragmentation of the god figure [78]. Dionysus/Bacchus had multifaceted meanings related to concepts of ecstasy, life and death, and being the god who died and was reborn, he was strongly linked to life beyond death [54,80]. Similarly, Etruscans often depicted bunches of grapes in the wall paintings of several tombs (*Tomba dell’Orco* (4th century BC), *Tomba dei Leopardi* (5th century BC), as an augural message for rebirth and joyful life in the afterlife [81]. The Christian religion incorporated pagan symbols, so the grapevine has persisted as a symbol of life and of wine as a symbol of blood through millennia [65,82].

When considering the figs, it is interesting to note that the wild fig in Rome was named *caprificus* (from *caper* = he-goat, and *ficus* = fig), and they handled branches of wild fig to favour the reproduction of the cultivated fig. Their infructescence are part of pollinator insects’ life cycle, and their presence is essential to guarantee the seed’s production [83,84]. We do not know if the Romans understood the complex cycle of fig fruits, but they did appear to understand the role of wild figs in pollination, and the assimilation with the he-goat, also sacred to the God Dionysus, is clear evidence of its significance of fertility. The figs also had a connection with God Mars in Roman mythology, as the father of the tween founders Romulus and Remus [39], and with the infant Zeus, who was fed by the goat Amalthea, the horns of which become symbols of abundance and fertility [85]. According to this, considering that most of the analysed festoons are represented hung to the horns of goats (frequently placed at the corners of altars), the symbolic link between goats and figs and that the latter were considered prestigious gifts for the deaths [86], the augural message of fertility and immortality assumes greater relevance.

## 4. Materials and Methods

### 4.1. Data Collection and Database Set Up 

First, we collected data on Roman carved festoons in artworks, checking the main museums and archaeological parks of Rome. The sources of data were *Palazzo Massimo*, *Palazzo Altemps*, the Diocletian Baths, and *Crypta Balbi*, all included in the *Museo Nazionale Romano*, the Vatican, Capitoline, *Galleria Borghese*, *Centrale Montemartini*, and *Fori Imperiali*, as well as museums and single monuments such as the Ara Pacis, Trajan column (in the Trajan *Fora)*, and Apollo Sosianus temple (next to Marcello Theatre), and finally the *Ostia Antica* and the *Appia Antica* archaeological parks.

We selected for chronology artworks dated between the I century BC and the III century AD, which are the most representative of the Roman culture before the influence of Christianity, and only in a few cases (6 festoons belonging to 3 artworks) the chronology was still debated or unknown. All of the artworks come from different locations in the modern city of Rome.

For each of the selected artworks, we collected photographs and information on the typology, provenance, chronology, context, and modern restorations that were included in a database. Moreover, to each artwork, we associated an identification number, indicating the museum (M-ID), the festoon (F-ID), and eventually the position of the festoon itself (C = centre; S = left; D = right). 

Furthermore, as the state of preservation of the sampled artworks varied from a very good to a low state, compromising the possibility of identifying the botanical species, we attributed to each festoon an evaluation of conservation status, according to this scale: (a) high level of integrity with well-preserved botanical elements; (b) medium level of integrity, and partially preserved diagnostic elements; (c) low level of integrity, and strongly compromised identification. All the information on the artworks and relative festoons (chronology, ID codes, festoon positions and their conservation status, museum inventory number (INV.), context and typology) are reported in Supplementary Data, S1a.

### 4.2. Plant Identification 

The identification of the botanical elements in the festoons was based on the procedure consolidated in previous studies [21,39,68,87]. Considering that the botanical represented elements were usually just a single part of the plant, such as fruits, leaves, flowers, or inflorescences, we considered the related specific morphological elements. For the leaves, we considered the shapes and composition (oval, truncate, elliptical, lanceolate, and linear; simple, or compound and edges), while for the flowers, we considered the number and shape of petals and the symmetry. Regarding fruits, we considered the typology, shape, and size overall, while for other reproductive structures, like the cones, we also considered the disposition and size of the scales. Then, all these morphological features were compared with the fresh plants in nature, looking for similarities or differences. We consulted online databases and herbaria considering the species that could be present during Roman times (e.g., Kew Royal Botanical Garden (https://powo.science.kew.org accessed on 25 February 2024), Dryades Home (https://dryades.units.it accessed on 30 March 2024), The World Flora Online (https://www.worldfloraonline.org accessed on 25 February 2024), The New York Botanical Garden Virtual Herbarium (https://sweetgum.nybg.org/science/ accessed on 25 February 2024), and the Global Biodiversity Information Facility (https://www.gbif.org accessed on 30 March 2024). Moreover, the resulting dataset was compared to iconographic elements from previous studies on plants in Roman artworks, including sculptural materials and frescoes, such as those from Pompeii [21,30,39,67]. We also considered the Italian Flora [88] and Dryades Project (https://dryades.units.it accessed on 30 March 2024) for the chorology of the plants. 

#### Symbolism of the Represented Plants

The symbolic significance of the plant representations was analysed by referring to the archaeological and historical literature, primarily considering classical Latin and Greek authors such as Theophrastus (371–287 BC) in the *Historia Plantarum*, Varro (116–27 BC) in the De Re Rustica, Lucretius (98/94–50/55 BC ) in the *De Rerum Natura*, Vitruvius (80/70–15 BC) in the *De Architectura*, Virgil (70–19 BC) in the Aeneid and Georgic, Horace (65–8 BC) in the Odes, Ovid (43 BC –17/18 AD) in the Metamorphosis, Pliny the Elder (23–79 AD) in the *Naturalis Historia*, and Dioscorides (40–90 AD) in *De Materia Medica*. Furthermore, we also examined studies on the symbolism of plant motifs in a wider area of ancient Euro–Mediterranean cultures and those in the Near East [11,39,48,51,55,89,90,91]. 

However, in some cases, the ancient sources could not be enough to understand the symbolism behind the identified plants. For this reason, we decided to apply the “associative method” proposed by Caneva [39], where the symbolic value of a plant can be hypothesised, taking into consideration its morphological and eco-physiological characteristics that give rise to its habitat, following traces of the human view of the plants that still remain in the etymology of the plant names. In this way, as the shapes were considered not casual and correlated with abstract ideas and religious values, forming the basement of the symbolic language in ancient cultures [24], we could reconstruct the associations between plants and beliefs in a similar way to how the ancients did. For each selected and analysed artefact, we also considered the typology of scenes and characters represented in association with the festoons (such as mythological, environmental, etc.), trying to understand if there was a clear symbolic association between the represented plants and certain scenes.

### 4.3. Festoon’s Structure and Organisation

To better understand the role of festoons in ancient societies, we analysed the variability of the composition of the botanical elements, searching for similarities or differences.

Botanical Diversity 

For the composition variability and to identify possible composition patterns, we analysed only the multi-species festoons to search for possible recurrences of groups or single elements in the festoons.

We analysed the structure of each festoon in particular, isolating every compositive botanical element and then creating unique sequences where the plant position is also collected. For each identified species, we associated a unique ID (i.e., *Ficus carica* = FI, *Punica granatum* = PU, etc.), and we attributed a specific colour, whereas, for the unidentifiable elements, we used a generic code of “IND”. 

To evaluate the floristic similarities between the festoons, we applied the Shannon –Wiener (1) and Evenness indexes (2). The Shannon index (H) is commonly used to quantify the biodiversity among ecological communities [92], defined as:

(1) $$H=\sum_{i=0}^{N}p_{i}\ln p_{i}$$

N represents the total number of species, and *p_i_* is the *i*th species. The index values range between 0 to infinity and proportionally to the number of species. Conversely, the Evenness index expresses the homogeneity degree of species distribution in the community [93], calculated as:

(2) $$E_{H}=H’/\mathrm{lnS}$$

H is the Shannon index and S represents the number of species. The index value ranges between 0 and 1.

These evaluations were also used to assess differences in the festoons, which could be related to their context, typology, and chronology, using a variance analysis (ANOVA) with a *p*-value < 0.05, followed by the Tukey’s test to determine which group means are significantly different from each other, through the program R 4.2.2. For each resulting group, we calculated the mean and one Standard Deviation (1SD) to evaluate the ranges between which the trends vary. 

Furthermore, to assess the similarities of species present in the festoons, based on their abundance in each festoon, their floristic patterns, and the recurrence of species in relation to the artwork typologies in which festoons are represented and how similar they are, we performed the PCA (Principal Components Analysis) and Cluster analysis using PAST 4.03 (PAleontological STatistics) software.

## 5. Conclusions

The analysis of the Roman festoons showed the presence of both mono-specific and multi-specific festoons, with a prevalence of foliage in the former and fruits in the latter. The first typology (mono-specific festoons) was exclusively composed of laurel or oak, which had strong connections with the Goddess Apollo and Jupiter/Zeus, respectively. When considering the multi-specific festoons, the identified taxa and their structure composition enhanced their augural messages of fertility, fecundity, and immortality, with a surprising absence of plants related to the concept of death. 

The 30 identified taxa showed different frequencies and a higher recurrence of species belonging to the genera *Laurus*, *Pyrus*, *Malus*, *Ficus*, *Punica*, *Vitis*, and *Quercus*. Some recurrent patterns, such as flowers at their centre and figs and vine grapes at the beginning and end of the festoons, suggest a non-casual disposition of the species. In the multi-specific festoons, they might have represented the primary elements on which the construction of festoons was based on. For most of the other identified species, it is not possible to link a recurrent pattern to the type or context of the artworks, even if *Pyrus communis* and *Ficus carica* appear to be more commonly associated with sarcophagi than with other types of artworks. Furthermore, the high plant diversity usually corresponds to an equal distribution of the botanical elements. 

The results of this research confirm the great attention the ancients paid to natural elements such as plants. Moreover, their reproduction was not only ornamental but also an instrument to highlight concepts and ideas. However, the loss of some elements related to the low integrity status of some of the analysed artworks makes future studies necessary in order to deepen our understanding of their botanical composition, tracing further messages hidden behind their representation.

## Figures and Tables

**Table 1:** The botanical taxa in the Roman festoons, their chorology and symbolic value. * = identified species coming from artworks characterised by modern restorations.

Plant Taxa Latin Names and (Family)	Represented Elements	Chorology	Diagnostic Traits	Symbolic Value
*Allium sativum* L. (Amaryllidaceae)	Bulb	Central–Asian	Bulb splits in “cloves”. Six petaled flowers with pointed apexes	Known since ancient times for its medical and superstitious values, assuming great relevance as a salvific plant [39,46]
*Anemone coronaria* L. cfr. (Ranunculaceae)	Flower	Steno–Medit.	Pentamerous flower with rayed symmetry, elliptic or rounded tepals, and a large floral disc. Possible confusion: *A. sylvestris*	Symbol of the rebirth of the earth given by rapid blooming. Named after the Greek nymph, who became a flower after she died, by Zephyr’s request to Venus [39,47,48]
*Arbutus unedo* L. (Ericaceae)	Fruit, leaves	Steno-Medit.	Short petiolate leaves with oblong lamina, acute apex, and serrated margin. Fruit as spherical berries long-stalked with grainy exocarp	Augural plant with positive symbolism, protective to malign spirits. Related to God Janus. In Greece, related to God Hermes [8,22,49]
* *Citrus x limon* (L.) Osbeck cfr. (Rutaceae)	Fruit	Asia (Hymalaya)	Oval/oblong fruits with pointed apexes	Its symbolism is controversial for ancient Rome due to its few traces in the Pompeian context [50]
*Convolvulus* cfr. *sepium* L. (*syn. Calystegia sepium* (L.) R.Br.) (Convolvulaceae)	Flower	Eurasian	Flower with radiate symmetry, funnel-shaped corolla, with five welded petals	Allusion to vegetative force. Dionysian element for its similarity with Ivy leaves and to the climbing habit [39]
*Corylus maxima* Mill. (Betulaceae)	Fruit	Europ.–Caucas.	Fruit covered entirely by two growing, pubescent, fringed floral bracts	Fertility and procreation symbol [51]
*Crataegus monogyna* Jacq. (Rosaceae)	Leaves in branches	Eurasian/ Paleotemp.	Oval-rhombic-edged leaves, with 1–4 incisions on each side, elongated lobes and parallel edges, and a truncated or wedge-shaped base Possible but unprobeable confusion with *Sorbus torminalis* and *Acer campestre*	Sacred to goddess Maia, who imposed chastity during May, the month of purifications. Dedicated to the goddess Flora. In Greece, altars were ornated with branches during weddings. [47,52]
*Cydonia oblonga* Mill. (Rosaceae)	Fruit, leaves	W-Asia	Pomoid-shaped fruit with typical ribs mainly at the bottom. Leaves ovate to oblong, with a slightly rounded apex	Associated with Hera and Aphrodite. Symbol of love and fertility. Identified as the fruit collected by Hercules in the Hesperides [22,39,53]
*Daphne laureola* L. cfr. (Thymelaeaceae)	Flower	Steno–Medit.	Small tetramerous flower with pointed petals	Explicit references to the value of this species are not present. Although the present name was given by Linnaeus, its resemblance to laurel may justify its association with Apollo even in ancient times.
*Ecballium elaterium* (L.) A. Rich. cfr. (Cucurbitaceae)	Flower	Euri.–Medit.	Rotated corolla divided into five lobes, pointed petals, and evident veins Possible confusion: *Bryonia* sp.	Allusion to the vegetative forces, and it can be considered a Dionysian plant [39]
*Ficus carica* L. (Moraceae)	Infructescence, leaves	Medit.–Turan.	Leaves with blade 3–5-lobed, broadly ovate in outline; margin serrate at the apex of the lobes and large central vein. Fruits represented at maturity with trimerous opening	Symbol of fecundity, linked to Dionysus and also to Mars. It is the sacred tree where Romulus and Remus found repair. Symbol of fertility for its fruits, which resemble male sexual attributes and female ones (when mature and partially open) [52,54]
*Hedera helix* L. (Araliaceae)	Fruit, leaves	Subatl./Submedit.	Ovate/rhomboidal leaves (flowering branches) or palmate-lobate (vegetative branches). Fruits are globular ovoid berries	Emblematic plants associated with Dionysus. Symbol of eternity as it is an evergreen plant [39,48,54,55]
*Juglans regia* L. (Fagaceae)	Fruit	W-Asia	Ovoid or globoid fruit with fibrous and fleshy shell (husk) which contains the woody endocarp	Symbol of fertility and abundance, for a fruit shape similar to men’s sexual attributes. Sacred to Jupiter [39,51,52]
*Laurus nobilis* L. (Lauraceae)	Flower, fruit, leaves	Steno–Medit	Alternate coriaceous leaves, elliptical or oblanceolate with undulating edges. Subsessile ovoid berries as fruits, tetrameric flowers	Main Apollonian emblem, symbol of sun and victory. Symbol of Roman emperors, which used laurel leaves for crowns. Symbol of peace and prosperity. [41,52,56]
*Malus* cfr. *domestica* (Suckow) Borkh. (Rosaceae)	Flower, fruit, leaves	Eurasian	Simple, oval leaves, slightly serrated, with acute apex. Fruit with globular shape, generally 5–9 cm in diameter (*M. domestica*) or 3–4 cm (*M. sylvestris*). Flower 5 petaled, with narrowed petal nail	Symbols of fertility and sexual passion, in Greece, were used as gifts at weddings. Immortality and power symbol, for its circular shape as the terrestrial globe [52,57,58]
*Mespilus germanica* L. (Rosaceae)	Fruit	Europ.	Globose fruit as a pome (2–3 cm) with persistent sepals giving a “hollow”.	In Greece, it was sacred to Cronos, and in Rome, to Saturn, fearsome gods but useful when needed. [52]
*Olea europaea* L. (Oleaceae)	Fruit, leaves	Steno–Medit.	Drupe ellipsoid to sub-globose; leaves opposite and leathery, blade lanceolate to elliptic, cuneate at the base	Sacred to Athena in Greece. Symbol of prosperity and peace in Rome. Olive crowns as a reward for heroes or in Olympic games [41,52,59]
*Papaver* cfr. *somniferum* L. (Papaveraceae)	Flower, fruit	Euri–Medit/ Subcosmop.	Tetramerous flowers with lobed margins and wavy. Fruits as sub-spheric capsules with rayed stigma Possible confusion: *P. rhoeas* or *P. setigerum*	Associated with the ancient gods of the night, sleep, and death in Greece. It was also associated with the goddess Demeter–Ceres for the abundance of seeds. [39,49,60,61]
*Philadelphus coronarius* L. (Hydrangeaceae)	Flower	Sud Europe.	Tetramerous flowers with petals of typical shape	We could not find explicit references to the value of species, but its ornamental value in doing garlands remains in the name.
*Phoenix dactylifera* L. (Arecaceae)	Fruit	Paleosubtrop.	Subcylindrical fruit variable in shape and size, with a persisting perigonium remaining on the base	Numerous symbols in ancient civilisations. In Egypt, it symbolised Fecundity and life. In Greco–Roman societies, it symbolised immortality, victory and glory. [47,49,62,63]
*Pinus* sp. (Pinaceae)	Cone, leaves	-	Cones with needle-shaped leaves	The plant and the female cone are symbols of fertility. Sacred to Poseidon, Dionysus, and to Great Mother Cybele and her son Attis. [24,48]
*Pinus pinea* L. (Pinaceae)	Cone, leaves	Euri–Medit.	Female cones with ovate-globular shape and needle-shaped leaves	
*Pinus* cfr. *halepensis* L. (Pinaceae)	Cone, leaves	Steno–Medit.	Elongated cones, conic or ovoid shape (5–12 × 3.5–4.5 cm) Possible confusion: *P. pinaster*	
*Pyrus* cfr. *communis* L. (Rosaceae)	Fruit, leaves	Eurasian	Ovate or elliptic leaves, notched at margins. Fruits “pyriform-shaped” measuring 5–16 cm (*P. communis*) and 1–4 cm (*P. pyraster*) with the persistent calyx remaining at the bottom	Sacred to Hera in Greece, its shape evoked the female body, associated with Aphrodite becoming a sexual and fecundity symbol. [52,54]
*Punica granatum* L. (Lythraceae)	Fruit	W-Asia	Typical roundish fruit (5–12 cm); when in an advanced stage of maturity, the fruit splits, revealing the seeds.	Symbol of fecundity, abundance and death. A Greek myth says that it was born from the blood of Dionysus; Romans used it to crown women during marriage as an abundance wish. [48,49,64]
*Quercus* cfr. *robur* L. (Fagaceae)	Fruit, leaves	Europ.–Caucas.	Oblong–ovate leaves, rounded lobes, divided no further than halfway to the midrib. Fruits (acorns) with a long peduncle, ovoid with a pointed tip. Hemispherical dome with rhombic scales which cover the achene by 1/4 to 1/2.	Emblem of Zeus–Jovis; considered the tree which supported the sky and the world’s axe. The fruits were considered fertilisers and aphrodisiacs. [41,48,54]
*Quercus* sp. (Fagaceae)	Fruits, leaves	-	Oblong–ovate leaves, rounded lobes. Fruits with a pointed tip and hemispherical dome with rhombic scales.	
*Rosa* sp. (Rosaceae)	Flower	-	Five petaled flowers, with obcuneate and bilobed petals, with +/− conic floral disc. Several wild roses can be possible	She was linked to the cosmic process of flowering. Attribute of Aphrodite, from where the goddess was born. [39,49]
*Triticum* sp. cfr (Poaceae)	Flower-fruits	Medit.–Turan.	Spike with rachis and spikelets at alternate nodes Possible confusion: *Hordeum* sp.	Symbol of abundance, fecundity, and fertility of the earth. It was associated with Demetra–Ceres. [49,60]
*Vitis vinifera* L. (Vitaceae)	Fruit, leaves	Euri-Medit/Caucas.	Leaves heart-shaped, strongly divided in 3–5 lobes with margins dented. Spheroidal or ellipsoidal berry fruit arranged in bunches	Typically associated with Bacchus and Dionysus. Used for wine production since Ancient Egypt. Linked to ideas of richness, fertility, rebirth, and life. [48,65]

**Table 2:** Variability in plant structure of Roman festoons according to their context and typology based on Shannon and Evenness index values. Df (Degrees of freedom); Sum sq (Sum of Squares); Mean sq (Mean square); F value (Ratio to determine the presence of significant differences between group means); Pr(>F)*—*(*p*-value).

Measure	Source	Df	Sum sq	Mean sq	F Value	Pr (>F)
Shannon	Context	2	4.108	2.054	8.487	0.00068 *
	Residuals	49	11.78	0.2404		
Evenness	Context	2	0.1339	0.06695	3.238	0.0478 *
	Residuals	49	1.0132	0.02068		
Shannon	Typology	3	1.174	0.3915	1.27	0.295
	Residuals	48	14.729	0.3068		
Evenness	Typology	3	0.1556	0.05187	2.511	0.0698
	Residuals	48	0.9915	0.02066		
Shannon	Chronology	5	7.576	1.5152	8.307	0 *
	Residuals	46	8.327	0.181		
Evenness	Chronology	5	0.3155	0.06309	3.49	0.00932 *
	Residuals	46	0.8317	0.01808		

* *p*-value < 0.05; context (Funerary, Sacral, Unknown); typology (Altars, Sarcophagi, Urns, Unknown); Chronology (I c. BC, I c. AD, I–II c. AD, III c. AD, Unknown).

**Table 3:** Post hoc Tukey test of Shannon and Evenness indexes of the festoons according to their context and typologies.

Context	p adj		Typology	p adj	
*Comparison*	*Shannon*	*Evenness*	*Comparison*	*Shannon*	*Evenness*
Sacral-Funerary	0.001 *	0.039 *	Frieze-Altar	0.646	0.447
Uknown-Funerary	0.705	1.000	Sarcophagus-Altar	0.679	0.285
Unknown-Sacral	**0.052** *	0.556	Urn-Altar	0.928	0.730
			Sarcophagus-Frieze	0.295	0.069 *
			Urn-Frieze	0.941	0.206
			Urn-Sarcophagus	0.520	0.943

* *p*-value < 0.05.

## Data Availability

Data are contained within the article and in the related Supplementary Data.

## Supplementary Materials

The following supporting information can be downloaded at: https://www.mdpi.com/article/10.3390/plants13192795/s1, Table S1a: general information of the artworks and festoons; S1b: ANOVA and Post hoc Tukey tests results for chronology.
